# Landscape Composition Affects Elements of Metacommunity Structure for Culicidae Across South-Eastern Illinois

**DOI:** 10.3389/fpubh.2022.872812

**Published:** 2022-05-03

**Authors:** Valeria Trivellone, Yanghui Cao, Millon Blackshear, Chang-Hyun Kim, Christopher Stone

**Affiliations:** Illinois Natural History Survey, Prairie Research Institute, University of Illinois at Urbana-Champaign, Champaign, IL, United States

**Keywords:** *Aedes albopictus*, biodiversity, Culicidae, land use, mosquito surveillance, species assemblage

## Abstract

The interplay among invasive alien vectors and the species assemblage of native potential vectors in areas of range expansion may affect the dynamics of pathogen transmission. In this study we investigate how *Aedes albopictus*, an invasive mosquito of considerable public health concern fits within mosquito communities at the edge of its range of distribution. This was addressed using a 2-year field survey of mosquitoes in south-eastern Illinois. We found that *Ae. albopictus* was more broadly distributed in this region than previously realized, with new occurrence records for nine counties. Abundance of this species varied strongly and peaked in locations of low-intermediate overall mosquito species richness. This differed from overall mosquito abundance, as well as abundance of another important vector, *Cx. pipiens*, for which the abundance-richness relationships were best described with power functions. Metacommunity analyses revealed that mosquito communities showed a non-random distribution with a Clementsian gradient, which suggests a pattern whereby distinct species assemblages are associated with specific habitats or environmental conditions. Land use was a significant underlying factor shaping mosquito community structure and species assemblages. Multivariate analyses showed that while *Ae. canadensis* and *Cx. pipiens* complex mosquitoes were associated with high and low proportions of wetlands in the environment, respectively, *Ae. albopictus* was most strongly associated with urban settlements. This work sheds light on landscape-level processes, such as niche differentiation driven by urban and agricultural development, structuring mosquito communities. We suggest that mosquito community assessments across habitats be incorporated as part of a One Health vector surveillance approach to aid in the goal of prediction and prevention of new and (re-)emerging vector-borne diseases.

## Introduction

The worldwide range expansion over recent decades of mosquito vectors of arthropod-borne viruses (arboviruses), such as *Aedes albopictus* and *Ae. japonicus*, poses a serious concern for public health (1, 2). The introduction and expansion of these container-breeding species can alter the epidemiology of arboviral diseases caused by native or introduced viruses and the interactions with other vectors (3). Examples include the recent outbreaks of chikungunya virus (*Chikungunya virus*, CHKV) in Italy and La Reunion (4, 5), and Zika virus (*Zika virus* genus *Flavivirus*, ZIKV) in Central Africa (6). In the United States, autochthonous transmission has been recorded for both these viruses (7–9). The spread of ZIKV through Central and South America and the morbidity associated with this virus (10, 11) intensified the public concerns about invasive mosquitoes and pathogens transmitted by them (10). In addition, these invasive alien mosquito species may co-occur with native known vectors serving as augmentative vector populations, ultimately increasing the incidence of arboviral diseases. For example, mixed populations of the native *Ae. triseriatus* and the exotic *Ae. albopictus* both contribute to the transmission of La Crosse virus (*La Crosse orthobunyavirus*, LACV), which has been the major cause of arboviral encephalitis among children in the United States (12–14).

Mosquito and arbovirus surveillance programs are a valuable tool to track viruses and define the risks for emerging diseases or increased virulence and persistence (15), and can benefit from being designed around an approach that targets the mosquito community as a whole (16, 17), including alien invasive species. However, the prevailing paradigm has historically been focused on single species of interest (18–20), posing challenges to implementing such programs (21). To develop comprehensive insight into the ways invasive mosquito vectors may affect vector-borne disease transmission patterns in newly invaded areas, it is important to not only understand the patterns and processes related to the distribution and abundance of invasive species, but to understand how they fit within or alter the broader mosquito community in newly invaded areas. One example of this relates to the paradigm of bridge vectors, species that due to their broader host utilization are unlikely to act as primary vectors driving transmission, but can play an important role in spreading pathogens between host species and increasing exposure in dead-end hosts such as humans (22). In the case of West Nile virus (genus *Flavivirus*, WNV), for instance, it has been suggested that in certain areas *Ae. albopictus* may play such a role (although this may be tempered by their apparent low feeding rate on avian species) (23), and understanding the extent to which they overlap within the landscape with primary vectors is an important component to understanding the risk (for humans) associated with their spread. Likewise, if competition occurs between possible vector species, understanding the consequences for co-occurrence is important in understanding how an invasive mosquito like *Ae. albopictus* may affect transmission of arboviruses such as WNV. Larval competition between *Ae. albopictus* and *Cx. pipiens*, for instance, has been documented and evidence suggests that *Ae. albopictus* is likely to outcompete *Cx. pipiens* in areas where they overlap and share larval development sites (7, 24–26). Additionally, a pressing question that was laid bare by the Zika epidemic was how an emerging arbovirus, once introduced into a new range, would be transmitted and which species of vectors would be involved in its transmission, leading to statistical predictive associations between vectors and viruses (27). Such novel associations between pathogens, hosts and vectors rely in part on the community ecology of vectors and understanding how invasive mosquitoes affect species assemblages (and thereby host-vector-pathogen networks) in different landscape contexts is thus likely to be an important component of predicting future risk.

The link between landscape composition and how it affects mosquito species assemblages and in turn the transmission intensity of vector-borne pathogens is an area of active study (28–30). A number of studies have explored the composition of mosquito communities in different habitats, for instance focusing on differences in species composition among different environments such as forest, field, or ecotone environments, or along urbanization gradients (18, 31–33). Relatively few studies however have assessed how invasive species such as *Ae. albopictus* take up residence within species assemblages and the landscape (32). Additionally, although we have studies that describe how mosquito species assemblages vary between different types of landscapes, there remains a lack of understanding regarding how (e.g., due to which processes) mosquito communities are assembled and in what manner they shift with changes in the landscape. Such questions require species distribution patterns to be addressed using a metacommunity framework which can statistically distinguish various possible distribution patterns, and can offer insight into the environmental components and processes that are primarily driving species assemblage structure among sites (34, 35). It has been suggested that processes involving entire communities at larger spatial scale may influence the dynamics of infectious diseases (36). Recently, Suzán et al. (37) proposed a conceptual model linking observed metacommunity structure with the distribution of potential and actual vectors or hosts across the landscape. According to this model, human modified landscapes will drive higher risk of emergence of diseases by favoring certain communities. They also hypothesized that nested metacommunities may support higher rates of transmission and pathogen prevalence across landscapes by supporting more competent vectors and related species. On the contrary, antinested structures (e.g., Clementsian), may interrupt pathogen transmission along a landscape gradient by filtering specific communities which share similar phylogenetic and ecological characteristics which determine vectorial capacity (37, 38). Understanding such patterns and processes in detail may aid in understanding how range expansion of an invasive vector might affect vector-borne disease transmission.

In response to concerns related to potential introduction of ZIKV into the United States, and a lack of systematic surveillance of *Aedes* species in Illinois prior to 2016, we initiated a 2-year *Aedes albopictus* surveillance project with the Illinois Department of Public Health. The objective of this project was to improve understanding of the current distribution and abundance of this invasive species in Illinois, at the current northward edge of this species' range in the U.S. (39).

Using mosquito surveillance data collected during this 2-year project (2016–2017) in south-eastern Illinois, we characterized mosquito community composition and diversity to improve understanding of the potential risk posed by invasive species in Illinois and spreading of potential vectors. We also assessed the underlying processes shaping the metacommunity structure. Specifically, we tested the hypothesis that mosquito assemblages are shaped by shared responses to a gradient of landscape-level variables defining the major boundaries among metacommunities.

## Materials and Methods

### Study Sites and Landscape Variables

A 2-year surveillance survey was conducted in 18 communities (hereafter locations: Albion, Benton, Cairo, Champaign, Charleston, Danville, Effingham, Fairfield, Lawrenceville, Marion, Marshall, Mattoon, Mt. Carmel, Mt. Vernon, Paris, Robinson, Salem, Tuscola) distributed in 17 counties in south-eastern Illinois. The locations were preselected with the intent to sample mosquito populations from a balance of counties from which *Ae. albopictus* either had or had not previously been reported, while including a diversity in the proportion of rural or semi-rural and urban habitats, which are the dominant landscape units in Illinois, and other natural habitat types. In each location, three sampling sites were selected in coordination with local public health districts. To characterize the habitat of each of the sampling sites, we made use of the Illinois Gap Analysis Program Land Cover Classification, 1999–2000 dataset (40). We used a 1 km buffer around each sampling site using the “gBuffer” function, and within this buffer calculated the proportion of land cover classified as either agricultural land, forested land, urban land, or wetland using the *sp, rgdal* and *rgeos* packages in R (41).

### Mosquito Collection and Identification

Mosquitoes were sampled using a BG-sentinel 2 trap to which a proprietary lure was added as an additional cue (Biogents, Regensburg, Germany). Dry ice was added to the traps in order to sample a broader proportion of the mosquito community. Three BG-sentinel traps were set for an approximately 24-h period every 2-weeks in each location between July and October in 2016 and 2017. The samples were transported to the laboratory in a cool box containing dry ice and were subsequently identified to species on a chill table using taxonomic key provided by (42). In cases where morphological identification was not possible with confidence (e.g., due to loss of identifying characters), specimens were identified to genus only. For instance, morphological identification of possible *Cx. pipiens*, hybrids with *Cx. quinquefasciatus*, as well as *Cx. restuans*, typically being recorded as *Culex* spp. This group of species includes taxa that are known as vectors of pathogens such as *Plasmodium* spp. (avian malaria), *Dirofilaria* spp. (filarioid helminths), and viruses such as Usutu, Sindbis, St. Louis encephalitis virus and WNV (43). Because our analyses of the mosquito communities use distinct species as variables, the occurrence of morphologically similar or cryptic species has to be taken into account. Therefore, to further clarify the genetic diversity and structure of these individuals, a subsample of 492 individuals (representing the relative proportion of the total individuals collected in each site) was selected for molecular analyses.

### DNA Extraction, PCR Amplification, and Sequencing

Total DNA was extracted from a single mosquito leg of each specimen using the Phire Tissue Direct PCR master mix kit (Thermo Fisher Scientific, Waltham, MA). The fragment of mitochondrial cytochrome C oxidase (COI) gene was amplified using the primer pair MTFN (5'-GGA TTT GGA AAT TGA TTA GTT CCT T-3') and MTRN (5'-AAA AAT TTT AAT TCC AGT TGG AAC AGC-3') (44). The amplicons were sequenced by the Keck Center at the University of Illinois at Urbana-Champaign. Each sequence read was BLAST searched at GenBank and the mosquito identification was selected if the sequence match was at least 97%. Sequences were edited by removing Ns at each end using a custom perl script and purging long successive Ns in the middle manually. To identify the samples, we performed TBLASTN searches against the standard nucleotide databases in NCBI under default settings except that only the top 10 hits were shown. Identification was confirmed only for those sequences with an identity value ≥ 97% and a matching length (query length ^*^ coverage) ≥ 600 bp compared with the reference sequences. Sequences matching more than three different species in the top 10 hits all showed low identity values therefore were excluded from further analyses. Sequences successfully identified as *Cx. pipiens* or matching both *Cx. pipiens* and *Cx. quinquefasciatus* but with low identity values or match length were aligned using PASTA v1.8.5 (45) under default settings. The alignment was checked and trimmed manually.

### Haplotype Analyses of *Culex pipiens* Complex

In order to analyze the genetic diversity, the 492 individuals of *Cx. pipiens* complex were grouped based on their location of collection and the haplotype analysis was carried out. The DnaSP6 program was used to identify haplotype diversity (46). All population genetic parameters, including the number of polymorphic (segregating) sites, haplotype number (Hap), haplotype diversity (Hd), nucleotide diversity (*n*), and neutrality statistic information, such as Tajima's D, were calculated. The NETWORK ver. 4.6.1.2 program (www.fluxus-engineering.com) was used to construct a median-joining haplotype network (47) with maximum parsimony calculation. Relationships among the haplotypes were depicted with PopART (Population Analysis with Reticulate Trees) v. 1.7 (http://popart.otago.ac.nz) (48).

### Statistical Analysis of Community of Culicidae

We first analyzed the functional forms describing the relationship between species richness (total number of species collected from a given location) and mosquito abundance per trap night of *Ae. albopictus, Culex pipiens*, and all mosquito species jointly. Specifically, we used the following functions: power (*y* = a^*^x^*b*^), logistic ($y=\frac{e^{\left(a+b^{*}x\right)}}{1-e^{\left(a+b^{*}x\right)}}$), ricker (*y* = a^*^x^*^e^(−^b^^*x^)^), Hollling type 3 ($y=\frac{a^{*}x^{2}}{b^{2}+x^{2}}$), shepherd ($\frac{a^{*}x}{b+x^{c}}$), and a linear function (*y* = a^*^x). These functions were chosen to represent a wide range of commonly encountered functional forms, and not necessarily related to the original reason they were developed. Abundance was modeled using a Poisson distribution and parameter values with 95% confidence intervals were obtained through maximum likelihood estimation using the “mle2” function from the *bbmle* package in R (49).

The metacommunity dataset includes 54 sampling sites each representing a local community of mosquitoes. We tested the hypothesis that mosquito metacommunity was non-random and the structure was assessed using the framework (EMS, Elements of Metacommunity Structure framework) proposed by (34) and (35). Three metrics were calculated: coherence (degree to which a pattern can be collapsed into a single dimension, ordination axis, or the significance of modularity, nestedness or randomness shown by the metacommunity), turnover (calculated if positive coherence is detected and is the turnover in species composition along a dimension or ordination axis. It measures the number of species replacements), and boundary clumping (which takes into account how the edges of species range boundaries are distributed along a dimension or an ordination axis). The metrics were calculated using the equiprobable rows and columns (“r00” method) and fixed–proportional null model (“r1” method, i.e., preserves the site (row) frequencies, but uses column marginal frequencies as probabilities of selecting species) and 1,000 simulations in the R package *metacom* (50). Differences in the coherence and turnover (z-values) between groups of sites based on landscape variables were tested using a Kruskal-Wallis test.

The sampling sites were classified based on their landscape similarity, the Silhouette Coefficient method was used to define the number of groups and K-means algorithm technique to divide the sampling sites into given K groups with nearest mean. For each landscape category (agriculture, forested, wetland and urban), between-group comparisons were performed using 2-tailed *t*-test or Yuen-Welch's test, as appropriate. The spatial autocorrelation of landscape variables was evaluated using the Moran's I coefficient (51) based on spatial weights matrix, by testing the null hypothesis that there is a random distribution of the landscape types in the whole study area. The significance was performed utilizing the Monte-Carlo permutation test after 999 permutations. If the MC observed value is greater than that expected by chance then similar landscape values are near to each other across space (positive spatial autocorrelation), while a significant negative value of MC indicates dissimilarity among neighboring values (negative spatial autocorrelation). Moran's I was computed using “moran.randtest” function in *adespatial* package.

The k-mean clustering algorithm was applied to the landscape matrix to segregate the sampling sites into k groups, with each of them being allocated in the closest group in the distance to its center (52). To define the parameter of optimal number of groups, we first calculated the silhouette score using “fviz_nbclust” and “kmeans” functions in *stats* and *factoextra* R packages. Once the clusters were calculated and each sampling site assigned, the Shapiro-Wilk test and F test statistic were applied to check if the variables were normally distributed and the homogeneity of variances. We tested the significance of the difference between groups for each variable using a Welch's *t*-test or Yuen's trimmed mean test (53) if homogeneity of variances or normality is violated.

Using the community species matrix, we calculated the sampling site scores derived from the primary reciprocal averaging axis (50, 54). These scores can be interpreted as a latent environmental gradient along which species distributions are structured. The non-parametric Spearman's rank correlation coefficient was used to test the association between sampling site scores and landscape values of each variable. To evaluate the variables most strongly associated with the variance of the observed communities, we applied redundancy analysis (RDA, a multiple linear regression model that accounts for multispecies response data and multiple explanatory variables) on the Hellinger-transformed abundance data and after removing singletons (species collected in 1 sampling site).

Finally, permutational multivariate analysis of variance (PERMANOVA); (55) with 999 permutations was used to test the effect of landscape units (agriculture, forested, wetlands and urban) on Culicidae relative abundance using the function “adonis” in the package *vegan*. The multivariate homogeneity of group dispersions (PERMDISP) was tested using the function “betadisper”.

All statistical analyses were conducted in R version 4.0.5 (41).

**Figure 1:**
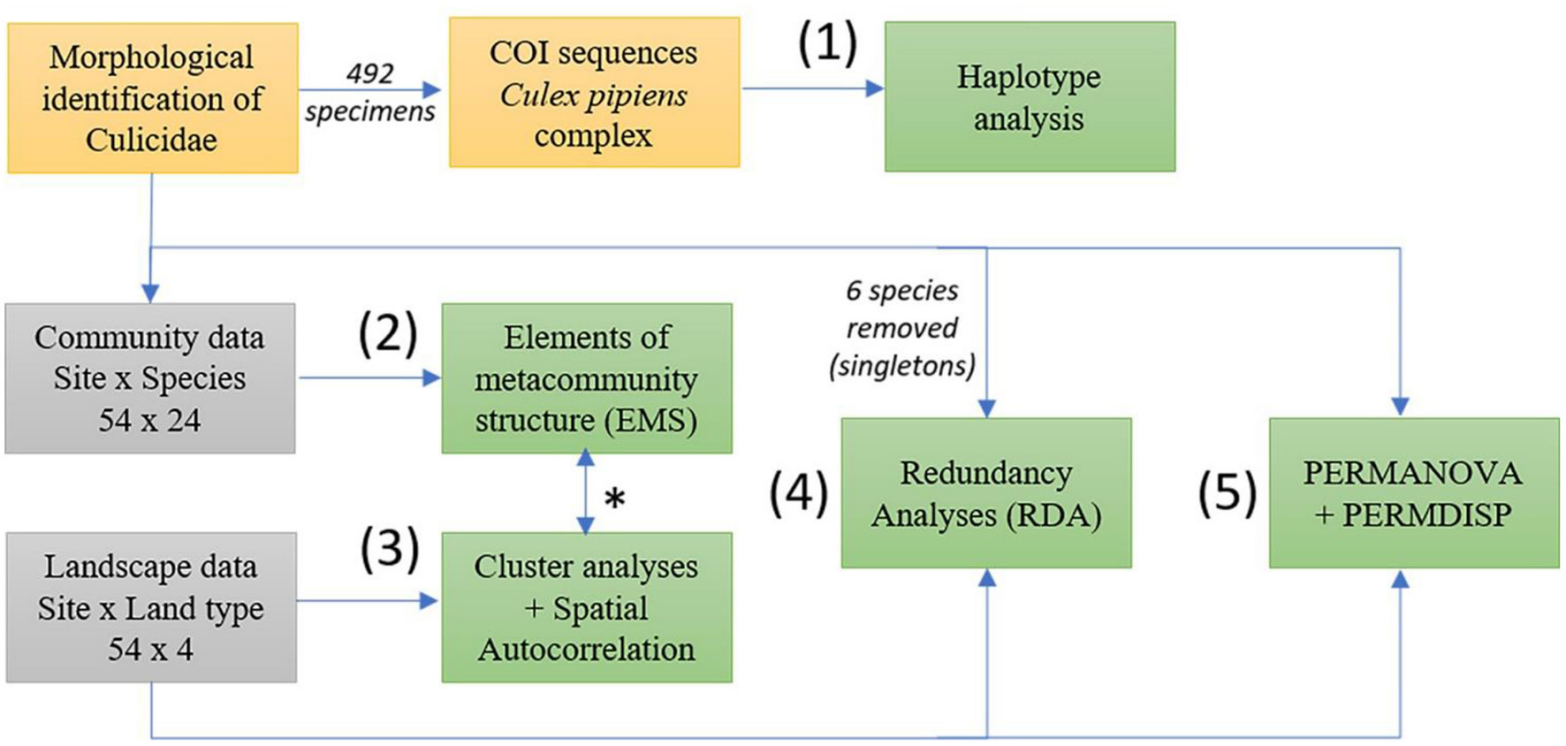
Scheme of the statistical analyses (boxes in green) used in this study. Mosquito and landscape data were collected in 54 sampling sites in south-eastern Illinois in 2016 and 2017. Individuals of Culicidae were identified using morphological characters and a subsample of 492 specimens belonging to *Culex pipiens* complex were further analyzed molecularly (boxes in yellow box), haplotype network analysis was applied to evaluate their genetic distance (1). Community data (box in gray) were used to identify metacommunity properties emerging from the observed structure (2). Landscape data (box in gray) informed cluster analyses, verify spatial autocorrelation of sites (3) and the correlation with sampling site scores from community data (*). Redundancy analysis with forward selection was performed to select the most important environmental variables that explain variation in the community matrices (4). PERMANOVA and PERMDISP were used to compare the variance and homogeneity of environmental and community distances between communities collected in different landscape units (5).

**Figure 2:**
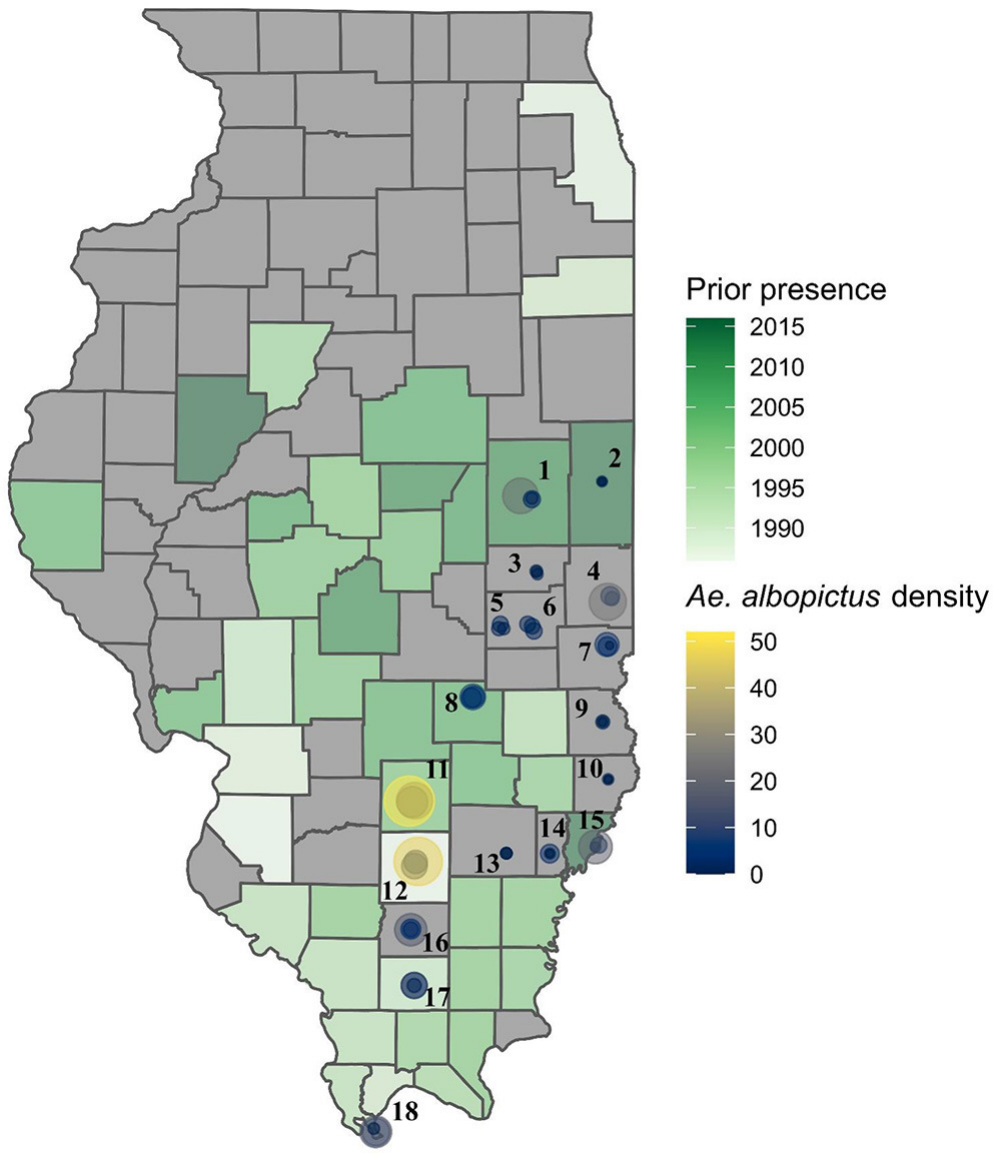
Sites within southern and eastern Illinois where mosquito surveys were performed in 2016 and 2017, first year of recorded occurrence of *Aedes albopictus* by county, and the mean density per trap per night for the sampling sites. Numbered sites: 1-Champaign; 2-Danville; 3-Tuscola; 4-Paris; 5-Mattoon; 6-Charleston; 7-Marshall; 8-Effingham; 9-Robinson; 10-Lawrenceville; 11-Salem; 12-Mt. Vernon; 13-Fairfield; 14-Albion; 15-Mt. Carmel; 16-Benton; 17-Marion; 18-Cairo.

An overview of the data analyses is presented in Figure 1 and the location of sampling sites in Figure 2.

## Results

### Species Composition, Abundance and Haplotypes

During the 2-years of surveys of known and potential vectors of arboviruses in Illinois a total of 24 species of Culicidae belonging to the genera *Aedes* (9 species), *Anopheles* (4), *Culex* (at least 3 species), *Orthopodomyia* (1), *Psorophora* (5), *Toxorhynchites* (1), and *Uranotaenia* (1) were collected (Supplementary Table 1). Four species (*Aedes albopictus, Ae. canadensis, Anopheles punctipennis*, and *Ae. vexans*) and the *Cx. pipiens* complex were present in most of the sites and most abundant (Supplementary Table 1, Supplementary Figure 1). Notably, *Ae. albopictus* was recorded from each county in which we sampled, including nine counties for which no prior occurrence record of this species existed (Figure 2), suggesting a wider patterns of spread and establishment of this invasive species throughout southern and central Illinois than previously realized.

**Figure 3:**
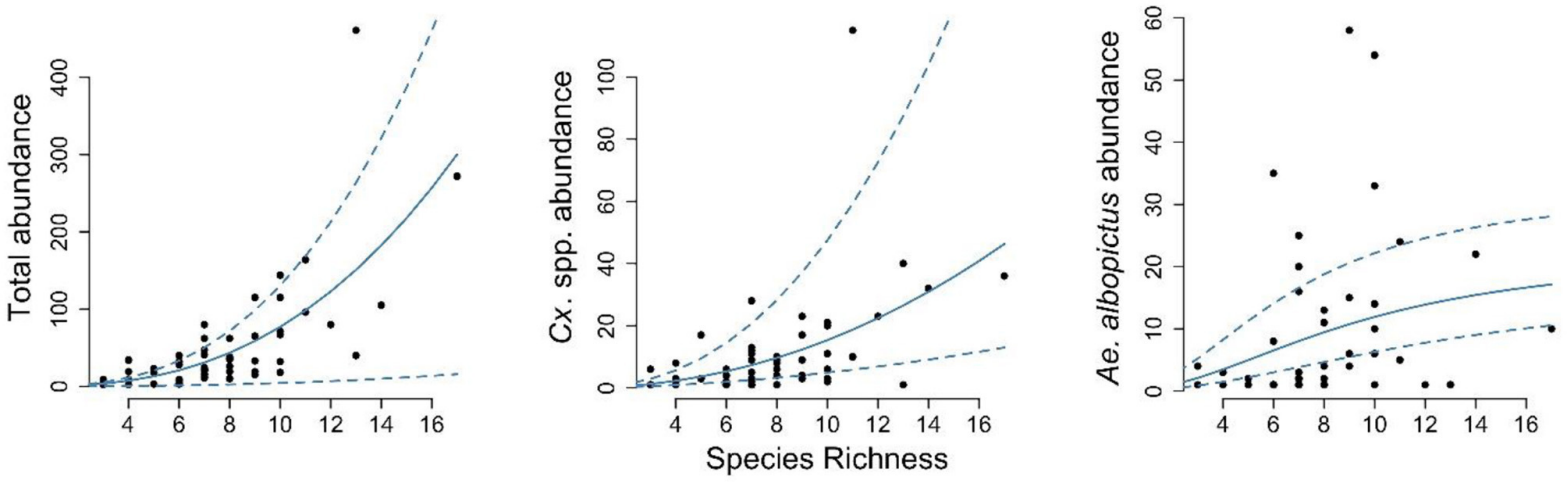
The relation between mosquito species richness and mosquito abundance. For each sampling site, the species richness over the entire season is compared the total mosquito abundance (left panel), the abundance of *Culex pipiens* complex (middle panel), and the abundance of *Aedes albopictus* (right panel). The solid blue lines represent the best fit, estimated using maximum likelihood, and the dashed lines the associated 95% confidence interval.

For both specific vectors of interest (*Ae. albopictus* and *Culex* spp.) and mosquito species combined there were large differences in average abundance between sampling sites. To understand how these differences relate to overall species richness, a measure of biodiversity, we explored these relations by fitting varying functional forms to the data. For total mosquito (all species combined) abundance, this relationship was best described by a power function, suggesting that in areas with more diverse mosquito communities, the overall abundance of mosquitoes is also greater (Figure 3).

The same functional form gave the best fit for the relation between species richness and *Culex* spp. (i.e., *Cx. pipiens* complex / *Cx. restuans*) abundance specifically. Greater numbers of *Cx. pipiens* were found in more diverse trap locations, and the relation between these factors was well-described by a similar power function. This indicates that mosquito species richness in an area could predict *Cx. pipiens* complex abundance. For *Ae. albopictus*, however, a different pattern was found, with the highest levels of *Ae. albopictus* abundance being associated with locations with moderate levels of species richness, and a Holling type III gave the best fit to these data (Figure 3).

A total of 492 individuals in the *Cx. pipiens* complex, collected in 2016 and 2017, were subjected to molecular analyses. The sequences were processed in BLASTN (GenBank) and showed an identity value higher than 97% and a matching length (query length × coverage) longer than 600 bp compared with the reference sequences belonging to species in *Cx. pipiens* complex. The final alignment of COI sequences represents 492 samples and contains 686 nucleotide positions. However, the best tree of the maximum likelihood analysis is not informative for the identification of the samples due to the overall low branch support values (tree not shown). Therefore, we further performed haplotype analyses. The statistical parsimony network calculated with TCS recovered one single network with 7 haplotypes (Ha1: 455 specimens, Ha2: 37, Ha3: 2, Ha4-Ha7: 1, Supplementary Table 2) which vary in six substitutions (Supplementary Figure 2). Haplotype diversity (Hd = 0.162 ± 0.022), nucleotide diversity (*n* = 0.0004), negative Tajima's D (−1.53) suggested bottleneck or a selective sweep. The most dominant mitochondrial COI haplotype in the analyzed material was Ha1, shared by 455 specimens of 492. Four haplotypes had one specimen only.

### Community of Culicidae and Landscape Variables

The community of Culicidae in central-south Illinois showed significant positive coherence, suggesting it is not well described by random or checkerboard processes. Additionally, turnover of species assemblages was significantly positive, and there was significant positive boundary clumping (Table 1). A pattern with these combinations of traits can be described as a Clementsian metacommunity, which indicates that the environmental drivers of community composition led to discrete groups of species which replace each other in a clumped or groupwise fashion (34). Because such patterns are often associated with landscape and ecotonal effects, rather than smooth gradients (e.g., related to temperature or elevation), we focused on land use for the remainder of the analysis.

**Table 1:** Elements of metacommunity structure (EMS) data for mosquitoes (Culicidae) collected in south-eastern Illinois.

		**r1**	**r00**
**Coherence**	df	51	51
	Abs	409	409
	z	−5.80	−41.55
	p	<0.0001	<0.0001
**Turnover**	Re	5,351	5,351
	z	2.72	2.74
	p	0.006	0.006
**Clumping**	Ml	9.04	9.03
	p	<0.0001	<0.0001
**Structure**		Clementsian	Clementsian

*Null model randomization method used: r1 (the species richness of a site is fixed and species ranges are filled based on their marginal probabilities) and r00 (equiprobable method with species richness and ranges having at least 1 entry)*.

**Figure 4:**
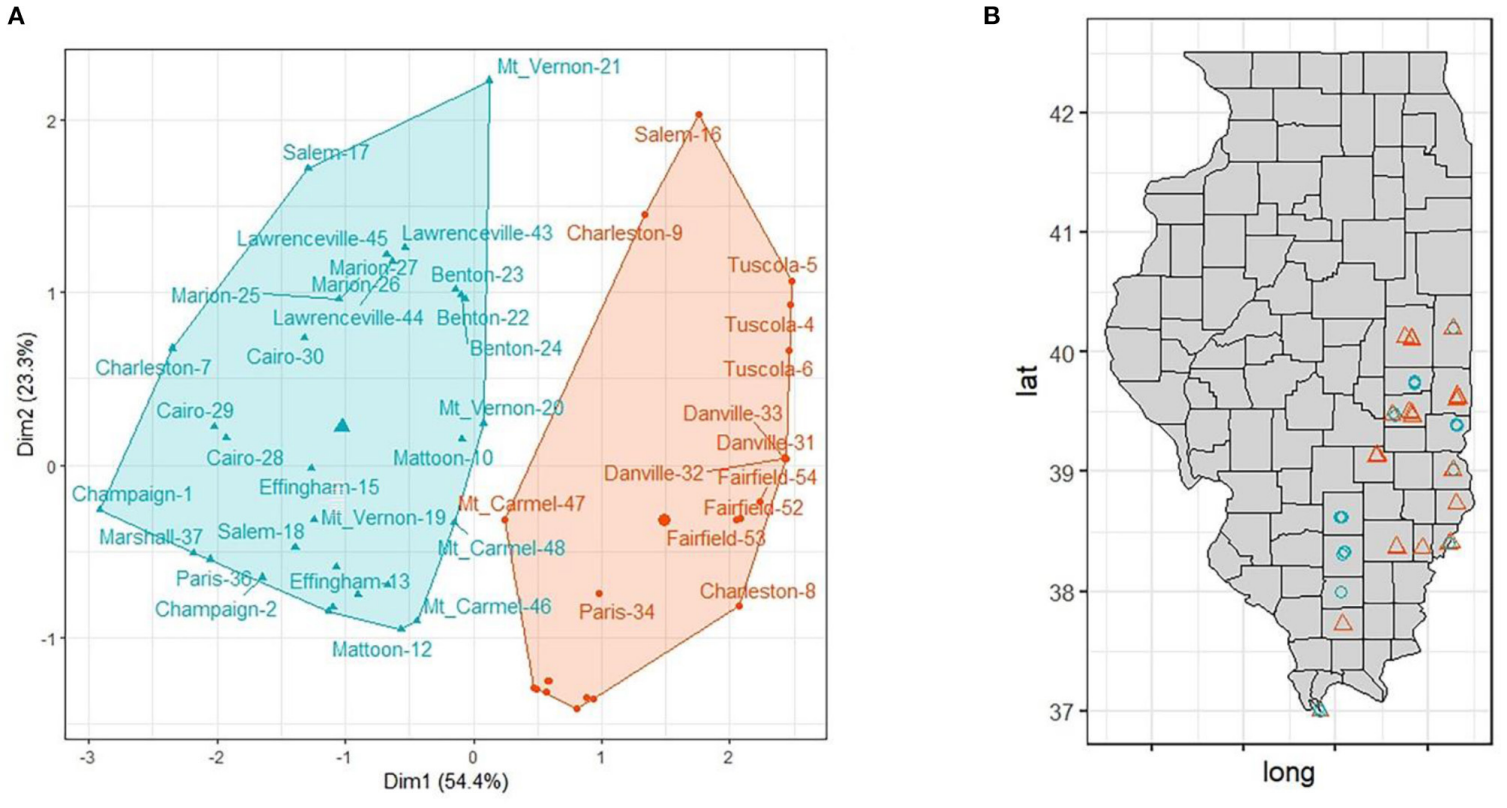
**(A)** Cluster analysis separated sampling sites in two main groups based on landscape variables (agriculture, wetlands, forested and urban). **(B)** Distribution of the of sampling sites in Illinois. Group 1 (*n* = 21): orange triangle; Group 2 (*n* = 33): blue circle.

We identified and characterized 2 main clusters of sampling sites with distinct landscape composition. The average silhouette metric peaked at two clusters and the k-means classification regrouped 21 (group 1) and 33 (group 2) sampling sites. Interestingly, almost all sampling sites belonging to the same location grouped together except for Salem-16 (in group 1), Charleston-7 (in group 2), Paris-36 (in group 2), Marshall-39 (in group 1), and Mt_Carmel-47 (in group 1) denoting a high degree of landscape variability among closest sampling sites (Figure 4). Group 1 included 33 sampling sites and was characterized by urban settlements, group 2 (21 sampling sites) was dominated by agricultural settlements (mainly annual crops, such as corn and soybean).

The comparison between the two groups showed that there is a significant difference among all variables (agriculture, urban and forested; *p* < 0.0001) except for wetland (*p* = 0.77) (Table 2).

**Table 2:** Two-sample *t*-test of the landscape variables analyzed for 54 sampling sites in Illinois.

**Variable**	**Group**	**Shapiro-Wilk**	**F-test**	**Welch two-sample *t*-test or Yuen *t*-test**				
		**Stat**.	**Sign**.	**Stat**.	**Sign**.	**Stat**.	**Sign**.	**df**
Agriculture	1	0.93	**0.12**	1.21	**0.62**	7.84	**8.8e-10**	42.44
	2	0.94	**0.07**					
Wetlands*	1	0.87	0.01	0.99	**0.98**	0.29	0.771	24.87
	2	0.89	0.003					
Urban*	1	0.87	0.007	0.99	**0.98**	−9.0	**9.82e-09**	21.43
	2	0.97	**0.41**					
Forested*	1	0.95	**0.26**	2.66	0.01	4.53	**3.0e-04**	16.83
	2	0.81	7e-5					

*The two groups of sampling sites are reported in Figures 4A,B. For Shapiro-Wilk test of normality p-values in bold (Sign.) indicate data normally distributed. For F-test of homogeneity of variance and t-test, p-values in bold (Sign.) indicate equality of the variances and difference in the averages of group 1 and 2.*

* *Yuen t-test applied when normality is violated*.

Spatial autocorrelation of each landscape variable was investigated using Moran's I and comparisons against null simulations. Overall, a significant positive spatial autocorrelation (Moran's I varying from 0.52 to 0.71, *p* < 0.001) was observed revealing that each landscape unit type was more clustered on the landscape than would be expected by chance (Supplementary Table 3).

When considering the correlation between sampling site scores (which summarize the mosquito community in each site) and each landscape variable there was a moderate positive correlation with agriculture and forested variables (rho = 0.26, *p* = 0.059 and rho = 0.27, *p* =0.049) and negative correlation with urban and wetlands (rho = −0.26, *p* = 0.056 and rho= −0.20, *p* = 0.141). To evaluate these associations in the multivariate space, we further investigated the effect of landscape variables using RDA.

**Figure 5:**
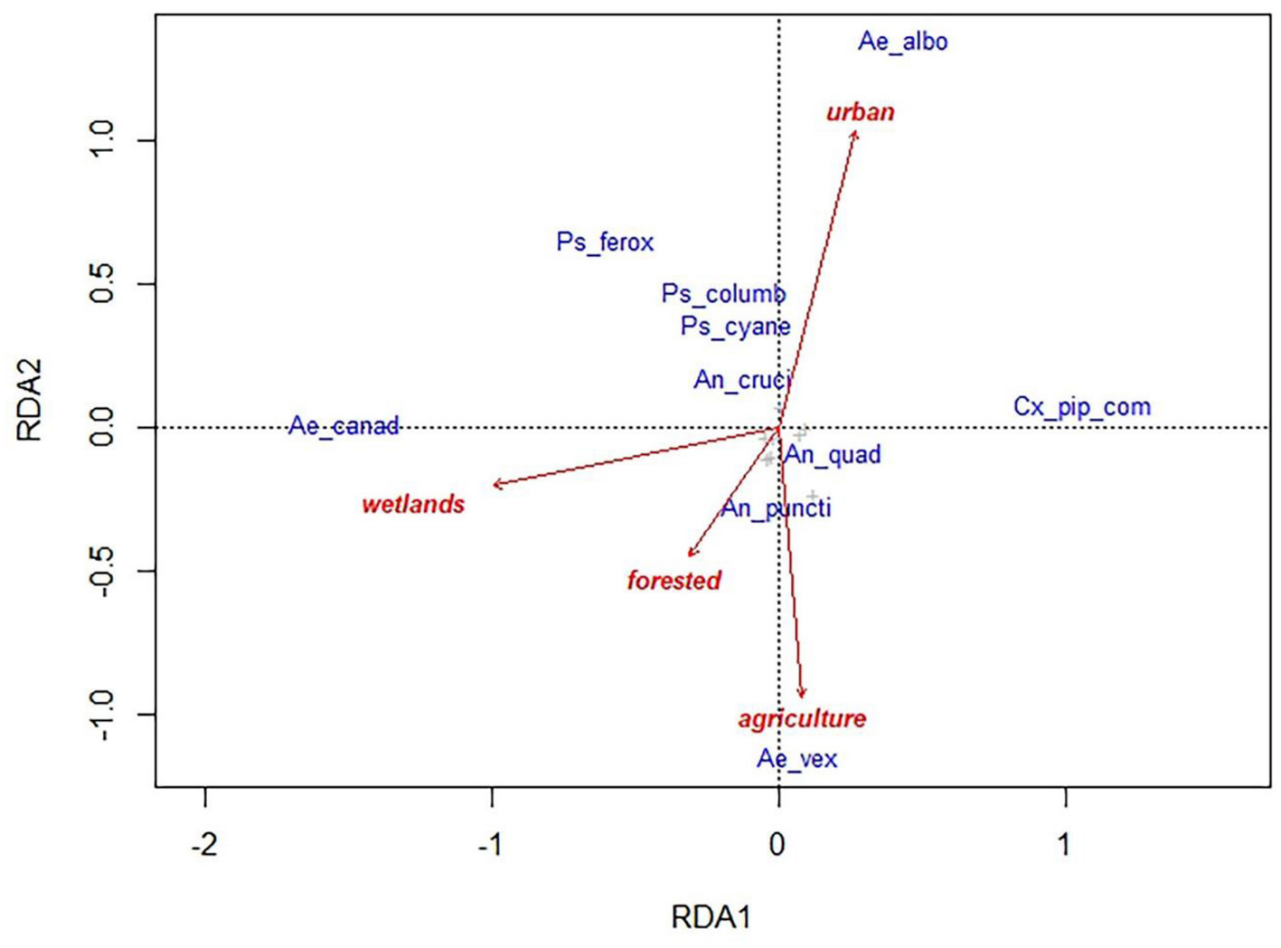
Redundancy analyses (RDA) ordination diagram for culicid species (in blue) and landscape variables (arrows in red) data collected in 54 sampling sites in Illinois. The full model explained 14% of the total variance, the first two constrained axis explained 7% (RDA1) and 6% (RDA2). Ae_canad, *Aedes canadensis*; Ae_albo, *Ae. albopictus*; Cx_pip_com, *Culex pipiens* complex; Ae_vex, *Ae. vexans*; Ps_ferox, *Psorophora ferox*; Ps_columb, *Ps. columbie*; Ps_cyane, *Ps. cyanescens*; An_cruci, *Anopheles crucians*; An_quad, *An. quadrimaculatus*; An_puncti, *An. punctipennis*.

The full RDA model for landscape variables was significant for the community composition of culicids (*p* = 0.02) explaining about 14% of the observed variation. The first constrained axis (RDA1) explains 7% of the variance, while the second (RDA2) explains 6%. The community of culicids across the different sampling sites were characterized by 4 species or group of species: *Ae. canadensis, Cx. pipiens* complex, *Ae. albopictus, Ae. vexans*. The first two are associated with the first RDA axis (−0.39 and 0.27 species scores, respectively) which is highly correlated with proportion of wetlands in the surrounding landscape. Higher values of wetlands favor presence and abundance of *Ae. canadensis*, whereas lower values favor *Cx. pipiens* complex. The second RDA axis showed positive relation between *Ae. albopictus* and higher values of urban settlements and between *Ae. vexans* and agriculture (0.32 and −0.28 species scores) (Figure 5).

The three factors: sampling sites, collecting period (4 months from July to October) and landscape, were considered altogether in the same permutational model (PERMANOVA). The community composition (a total of 24 species excluding singletons) of culicids was significantly affected by collecting period (*p* = 0.001) and landscape (*p* = 0.005), while the interaction between factors was not significant (Table 3). Analyses of the homogeneity of variance (PERMDISP) showed significant differences in community dispersion among farthest sampling sites (*p* = 0.019; Table 3). The difference between collecting periods (all combinations between months) and between landscape cluster groups (1 and 2) was not significant (Table 3). Because one of the assumptions for PERMANOVA, i.e., homogeneous dispersion, is fulfilled for periods and landscape, we can infer that the effect of these two variables on communities of culicids is reliable and not an artifact of heterogeneous dispersions.

**Table 3:** PERMANOVA and PERMDISP analysis of the effect of between groups of farthest sampling sites (3 “rep” for each city), collecting period (4 “periods” - from July to October), and landscape (cluster group 1 and 2 “land”) on community composition of Culicidae collected in central-south Illinois in 2016–2017.

**Source of Variation**	**Df**	**SS**	**MS**	**Pseudo F**	*p* (Perm)*	**EV (%)**
**PERMANOVA**						
*rep*	2	0.66	0.33	1.07	ns	1.2
*periods*	3	4.52	1.51	4.91	**0.001**	8.0
*land*	1	2.43	2.43	7.91	**0.005**	4.3
*rep* * *land*	2	0.46	0.23	0.75	ns	0.8
*periods* * *land*	3	0.83	0.27	0.90	ns	1.5
rep*periods	6	1.32	0.22	0.72	ns	2.3
*rep* * *periods* * *land*	6	1.21	0.20	0.66	ns	2.1
Residuals	147	45.14	0.31			79.8
Total	170	56.56				100
**PERMDISP**						
*rep*	2	0.06	0.03	3.90	**0.019**	
Total	168	1.41	0.01			
B-A					ns	
C-A					**0.03**	
C-B					ns	
*periods*	3	0.03	0.01	0.69	ns	
Total	167	2.48	0.01			
August-July					ns	
September-July					ns	
October-July					ns	
September-August					ns	
October-August					ns	
October-September					ns	
*land*	1	0.03	0.03	3.20	ns	
Total	169	1.70	0.01			
1–2					ns	

*Number of permutations: 999. The factor city was included as random factor. Df, degrees of freedom; SS, sum of squares; MS, mean squares; Pseudo F, F value by permutation; p (perm), p-value by permutation; EV, variance explained.*

* *, boldface indicates statistical significance at p < 0.05; ns, not significant*.

## Discussion

There is a growing recognition for the need to anticipate, prepare for, and prevent emerging infectious diseases (EIDs) through action programs and initiatives such as those based on One Health approaches (56–58). The main thrust is to use a holistic method to consider the links among human, plant, animal health and the environment, with a focus on understanding how anthropogenic pressures on the environment (e.g., land use change or urbanization) shape the threat of EIDs. A recent formalized strategic protocol (DAMA, Document-Assess-Monitor-Act) to prevent EIDs proposed actionable information to anticipate outbreaks (59), and assessing the risk space requires the evaluation of the interfaces among landscape types (17). In the context of mosquito-borne diseases, information about the influence of landscape type on community composition and the prevalence of mosquito species of particular interest are of pivotal importance to predict emergence and spread of zoonotic diseases. The diversity and species composition of vector communities can play an important role in shaping the transmission intensity of vector-borne pathogens (30, 60, 61), yet our understanding of the environmental factors that shape mosquito communities is still relatively limited. This is particularly true when considering how invasive species fit within existing assemblages in areas of range expansion, yet such situations may be particularly important when considering potential emergence of novel or neglected pathogens.

To better understand the patterns and processes behind the overall mosquito metacommunity in our study, i.e., how species assemblages vary among sites, we employed the elements of metacommunity structure analysis framework. This suggests that these assemblages are Clementsian, i.e specific groups of species characterize discrete units of landscape types. One of the elements, coherence, which was significant and positive, suggests that these assemblages are distinctly non-random, and are not well-described as checkerboard patterns, which could occur if competitive interactions dominate. They are also characterized by a significant positive amount of turnover, which suggests that there are unique, differing assemblages throughout the landscape, rather than nested subsets (which would point toward, for instance, a process whereby a full community exists in pristine habitats, from which species are lost as the habitat degrades). Finally, the community is described as having clumped boundaries, suggesting that small groups of species change or are lost with changes in the environment. A gradual loss or turnover of species is typically associated with environmental gradients such as temperature or elevation as shown for other communities [e.g., (62)], which does not appear to be the main driver of the communities in this region. Instead, such clumped turnover can be associated with changes in land use and in particular with ecotonal landscapes, i.e., interfaces among habitat types (35). Due to this, as well as significant correlation between the ordinated site scores and different land use classes, our results further indicate metacommunities shaped by habitat preferences with species in each community showing similar responses to environmental gradients that replace each other across space. Ecotones have previously been associated with high abundance of *Cx. tarsalis*, and can be used by mosquitoes as flight paths and for host seeking (63, 64). Such interfaces between distinct land use types can thus both mark transitions between distinct habitats harboring their own communities, but also provide a unique habitat within which specific mosquito assemblages can be associated (18). The consideration of habitat interfaces and fragmentation (potentially associated with agricultural use or urban development) was not incorporated directly in this study but is a promising direction for future research.

Our PERMANOVA and RDA analyses highlighted that different species were most significantly associated with different proportions of land use. *Aedes canadensis* was associated with areas with a higher degree of land characterized as wetlands, while the *Cx. pipiens* complex was associated with environments with low levels of wetlands. *Aedes vexans* was most strongly associated with agricultural areas, while *Ae. albopictus* was most associated with urban or developed lands. The latter fits with habitat associations from single species evaluations [e.g., (65)].

Other studies that have investigated the effect of landscape on community composition of Culicidae have likewise found that landscape type affects the structure of mosquito communities (66). In the same study, a non-significant effect of sampling period was reported which was probably due to the climatic conditions of the subtropical zone. In our study the sampling periods have a significant effect suggesting the importance for climatic factors to be included in prediction models in areas where constraints such as temperature fluctuations may affect growth rates of populations.

It has been suggested that the type of discrete communities associated with a Clementsian metacommunity structure could limit spread of pathogens over the landscape due to a partitioning of competent reservoir hosts or vectors (37). For vector-borne diseases, this prediction remains to be tested and was beyond the scope of the current study. Limiting transmission to specific habitats or seasons is most likely to be relevant for vector-borne pathogen systems that rely on multiple amplifying and possible bridge vector species. Seasonal changes in metacommunity structure are also likely to be of importance, as they may further partition competent vectors, and the importance of bridge vector species in terms of risk of exposure depends on when their seasonal peak occurs in relation to that of amplifying vectors.

A limitation to the use of metacommunity analysis based on species (co-)occurrence is that variation in population size or abundance of different species is not considered directly. Two important vectors, *Ae. albopictus*, and *Cx. pipiens*, for instance, were broadly distributed, but the way species-specific abundance relies on the species assemblage in which they are embedded could provide additional important insights.

Our analysis of mosquito abundance, i.e., all mosquito species combined, in relation to species richness reveals that it is well-described by a power function. Such a relationship could imply that environments with greater abundance of individual species result in a lower loss or local extinction (e.g., as a result of the habitat representing a larger continuous whole or placed in the landscape to be more receptive of introductions from nearby populations) of species and therefore a higher richness or could be the result of correlation with another factor (e.g., habitat productivity) (67). Interestingly, this relationship also held true for *Cx. pipiens* complex abundance, which increased according to a power function with overall species richness. For *Ae. albopictus*, abundance peaked at intermediate levels of species richness, suggesting that its population size is regulated through a different mechanism or that it thrives in distinct, more depauperate habitats. The latter has been reported elsewhere, for instance suggesting that suburban developments with age shift toward mosquito communities that are both dominated by *Ae. albopictus* and less diverse overall (32). Land cover disruption has been suggested as an important driver of the colonization of this species as well the biotic homogenization of the entire community of Culicidae (68). We were not able to test or differentiate between different processes that could lead to such outcomes [e.g., competitive displacement and competition between species (26, 69)], or factors that could affect the extent to which this occurs (e.g., seasonal shifting, microhabitat segregation).

The initial motivation for this study was the discovery and understanding the spatial spread, over other species of native Culicidae, of *Ae. albopictus* (Asian tiger mosquito), one of the most successful invasive species whose worldwide spread comes paired with significant concerns for human health (70). Our study revealed that *Ae. albopictus* was ubiquitously present in all communities in the current study, and notably was recorded in each county included in the surveillance effort, including nine for which prior to 2016 no occurrence record of this species in the state existed (39, 71) (Swanson, J., pers. comm.). These findings suggest either the continued expansion of this species' distribution throughout the state, or alternatively that it was already established but previously overlooked (e.g., due to use of traps that are less likely to pick up this species). We can infer that this species is now established throughout much of the southern half of the state. As *Ae. albopictus* is a highly competent vector for a wide range of arboviruses, the risk of arboviral outbreaks continues to pose a threat. West Nile virus is ubiquitous in Illinois and *Ae. albopictus* is, at least in the lab, a competent vector (72). It may also serve as a bridge vector for *Eastern equine encephalitis virus* (EEE) and serve as a vector for LACV (73). Understanding whether and how the expansion and increase of *Ae. albopictus* populations changes the landscape of arboviral transmission risk is thus an area in continuing need of research. Understanding the implications for vector control is likewise important. For instance, it was shown that co-occurring populations of *Ae. triseriatus* and *Ae. albopictus* cover a wider spatial and temporal range which makes targeted control measures more difficult (74).

Besides *Ae. albopictus*, the survey results indicated that at least 4 native species, *Ae. vexans, Ae. canadensis, An. punctipennis* and *Cx. pipiens* complex, were ubiquitously present in the surveyed communities. Although typically thought of as a nuisance biting mosquito, at least one study has shown that field-collected *Ae. vexans* are capable of transmitting Zika virus (75), and may also serve, in certain situations, as a bridge vector of WNV (22). As these species (and others, such as *Ae. japonicus* and *Ae. triseriatus*) can interact with *Ae. albopictus*, it will be important to understand whether their role in transmission of WNV, LACV, or other arboviruses, is altered as a result of their ecological interactions. This points to the importance of baseline information on likely co-occurrence of different species along a landscape gradient.

In our study, preferences for landscape types (Figure 5) showed that the four species have specific preferences for different habitats with partial ecological niche overlap. Similar results were obtained in other studies where anthropogenic habitats explained the presence of *Cx. quinquefascaitus* (76) and may be used as predictors to find WNV-positive mosquitoes (77).

A challenge in assessments of species assemblages relates to the existence of cryptic, co-existing species and hybrids. The *Cx. pipiens* complex in southern Illinois has previously been shown to fall within the region where *Cx. pipiens* and *Cx. pipiens*–*Cx. quinquefasciatus* hybrids co-occur (78). Individuals of the complex are very similar morphologically, and mitochondrial and nuclear markers are often used to characterize genetic diversity of cryptic species [e.g., (79)]. Additionally, another WNV vector present in this area, *Cx. restuans*, can be challenging to distinguish morphologically (80) from *Cx. pipiens* and are often (as we did here) reported as *Culex* spp., rather than as distinct species. To obtain insight into the genetic diversity within these samples, we sequenced a barcode region for a subset of specimens that were only identified to genus level. Specimens analyzed in this study showed a low genetic variability as noted in previous studies conducted in other countries (81, 82). Notably, all specimens analyzed in BLASTN database matched *Cx. pipiens* complex, suggesting that we may have not collected specimens of *Cx. restuans* or a very low presence within these samples. Whether this reflects the true composition in these environments, reflects the time of season of the collections (not starting until July) due to the earlier seasonal peak in *Cx. restuans* (83), or a bias related to the type of traps used is unclear. Our analysis identified one single haplotype that was dominant throughout the region, and a second, less common, haplotype (37 specimens out of 492) that was primarily found in the southernmost tip of the state, in Cairo, IL. We may speculate these specimens belong to *Cx. quinquefasciatus* as they match a haplotype identified as such in an earlier study, although further research evaluating the structure of this complex in Illinois in relation to land use is needed (84). One explanation for the dominance of one single haplotype could be related to the single sampling technique used or to the low coverage of natural habitats [e.g., (85)]. The reliance on BG Sentinel traps is also likely to have limited the species diversity in our samples by reducing the likelihood of sampling mosquito species that are not likely to be picked up in these traps (although the addition of dry ice as a CO_2_ source may have reduced this bias), and for future studies the use of multiple trap types to allow for sampling a broader diversity of the mosquito community is encouraged.

To conclude, an important invasive mosquito species, *Ae. albopictus*, is more widespread in Illinois than was previously realized. Using a number of analyses related to mosquito community ecology, we show that this species is particularly common in urban environments, and in areas with low to moderate species diversity. Questions that remain are for instance whether competition for resources in such urban environments negatively affects other mosquito species, and thereby shifts or changes the assemblage as this species invades. Information on species assemblages and how they change with land use inform potential risk assessments [e.g., (17)] for introductions of novel pathogens or (re-) emergence of diseases, and we recommend that such assessments of mosquito species assemblages and diversity be considered within One Health surveillance approaches.

## Data Availability Statement

The dataset of the cytochrome c oxidase subunit I (COI) mitochondrial gene sequences and the original final sequence alignment used in the haplotype analyses for this study can be found in the Illinois Data Bank (DOI: 10.13012/B2IDB-5032907_V1). Other data presented in the study are included in the article/Supplementary Material.

## Author Contributions

VT and CS drafted the paper, developed the data analysis, and led the writing of the manuscript. VT performed the statistical multivariate analyses and haplotype analyses. C-HK designed the field study. All authors critically reviewed, discussed, modified the manuscript, contributed to the article, and approved the submitted version.

## Funding

This study was funded by the State of Illinois Used Tire Management and Emergency Public Health funds and through an agreement with the Illinois Department of Public Health. Points of view or opinions expressed in this document are those of the authors and do not necessarily represent the official position or policies of the Illinois Department of Public Health.

## Conflict of Interest

The authors declare that the research was conducted in the absence of any commercial or financial relationships that could be construed as a potential conflict of interest.

## Publisher's Note

All claims expressed in this article are solely those of the authors and do not necessarily represent those of their affiliated organizations, or those of the publisher, the editors and the reviewers. Any product that may be evaluated in this article, or claim that may be made by its manufacturer, is not guaranteed or endorsed by the publisher.
