# Supplementary material for: Landscape Composition Affects Elements of Metacommunity Structure for Culicidae Across South-Eastern Illinois
Source: Front Public Health. 2022 May 3;10:872812. doi: 10.3389/fpubh.2022.872812 (PMC9110776; doi:10.3389/fpubh.2022.872812)

**Supplementary Figure 1.** Ordinated matrix of species occurrence (with embedded absences filled in) for sites in 18 counties of Illinois. Three sampling sites were used per county, consisting of BG sentinel traps baited with a lure and dry ice, run over the course of the summer of 2016 and 2017.

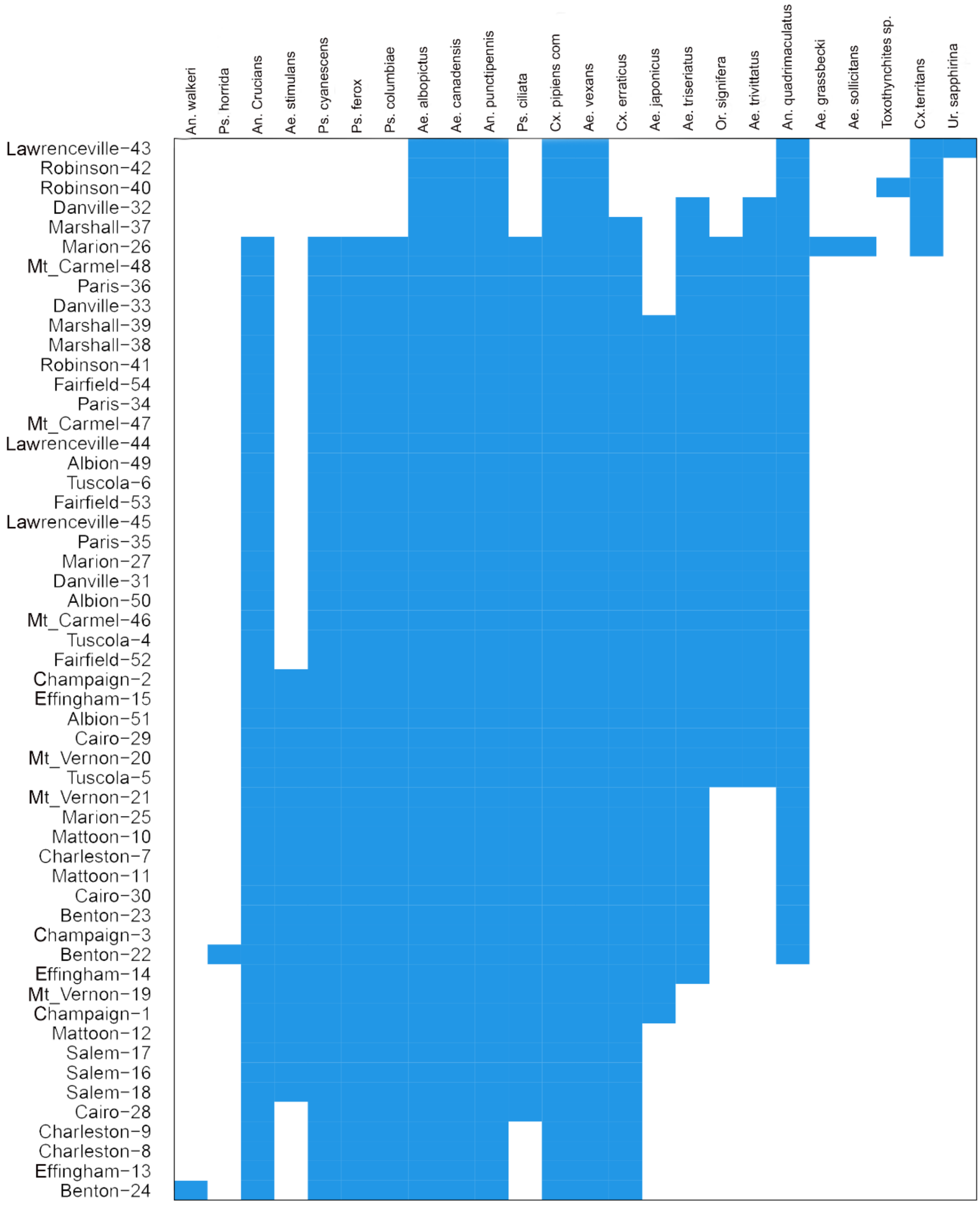

**Supplementary Figure 2.**

Median-joining network of mtCOI haplotypes obtained from 492 specimens in the *Culex pipiens* complex collected in 14 sampling sites (coded in colour, see Legend) in south-eastern Illinois. After the haplotype code (Ha1-HA7) the number parenthesis refers to the total of specimens with a particular haplotype. Lines between the circles are mutational steps.

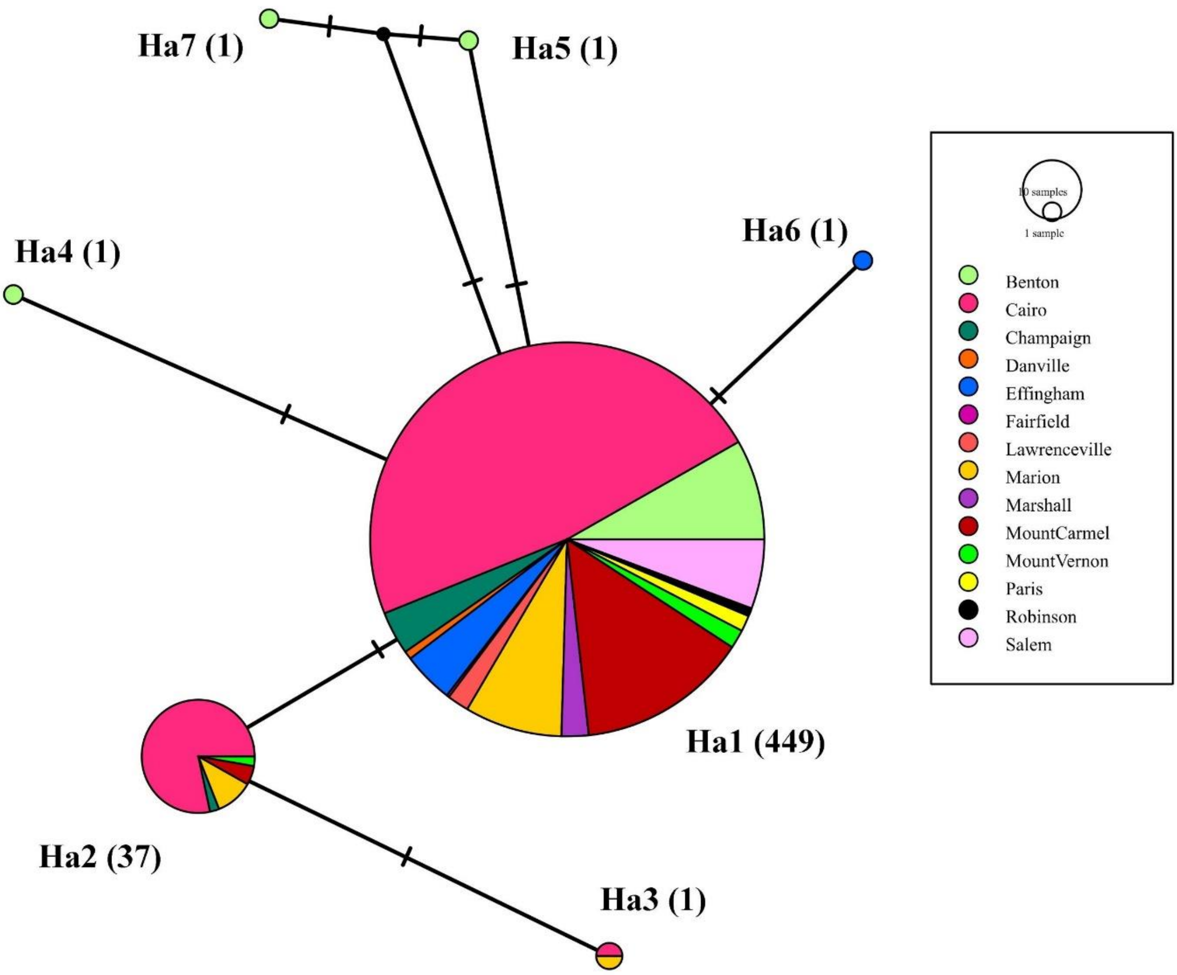

Supplement: Supplementary file 1 [file Data_Sheet_1.pdf]
